# Convergent and Discriminant Validity of the Barthel Index and the EQ-5D-3L When Used on Older People in a Rehabilitation Setting

**DOI:** 10.3390/ijerph181910314

**Published:** 2021-09-30

**Authors:** Billingsley Kaambwa, Norma B. Bulamu, Christine Mpundu-Kaambwa, Raymond Oppong

**Affiliations:** 1 Health Economics, College of Medicine and Public Health, Bedford Park Campus, Flinders University, Sturt Road, Bedford Park 5042, Australia; norma.bulamu@flinders.edu.au; 2 Health and Social Care Economics Group, College of Nursing and Health Sciences, Bedford Park Campus, Flinders University, Sturt Road, Bedford Park 5042, Australia; christine.mpundu-kaambwa@flinders.edu.au; 3 Health Economics Unit, Institute of Applied Health Research, University of Birmingham, Birmingham B15 2TT, UK; r.a.oppong@bham.ac.uk

**Keywords:** Barthel Index, ED-5D-3L, construct validity, discriminant validity, factor analysis, preference-based

## Abstract

This study compares the empirical performance of a commonly used functional-status measure, the Barthel Index (BI), to that of a widely used generic preference-based instrument, the EuroQoL-5-Dimensions 3 Level (EQ-5D-3L), in older people. Data from older people receiving rehabilitation services were used to test the validity of the BI and EQ-5D-3L. Convergent validity was investigated using Spearman’s correlation, exploratory factor analysis (EFA), scatter plots, Krippendorff’s alpha and modified Bland-Altman plots. Discriminant validity was examined using Kruskal Wallis tests, ceiling effects and EFA. A total of 1690 participants were included in the analysis. The BI total and EQ-5D-3L utility scores showed moderate correlation (r = 0.51; Krippendorff’s alpha = 0.52). Kendall’s Tau-B correlations between BI items and EQ-5D-3L dimensions measuring the same construct were weak to moderate (0.05 ≤ absolute r ≤ 0.54). In the EFA, some BI items cross-loaded onto the same factors as EQ-5D-3L dimensions, suggesting that the instruments were interrelated. The BI, however, focuses more on physical functioning, while the EQ-5D-3L measures broader wellbeing concepts. Both instruments showed good discriminant validity and would therefore be equally valuable for measuring subgroup differences. Researchers should consider using the BI in rehabilitation to capture more physical functioning-specific constructs not measured by the EQ-5D-3L.

## 1. Introduction

Health-related quality of life (HRQoL) and functional status are interrelated concepts that reflect important outcomes for older people receiving rehabilitation interventions. HRQoL indicates a person’s overall health status applicable in different contexts, including clinical studies, health care economic evaluations and population health surveys [1]. On the other hand, functional status and preventing functional decline, in particular, is seen as one of the prime objectives of health care for older people during rehabilitation [2], with a reduction in physical function linked to loss of independence, the need for hospital and long-term nursing-home care, premature death and quality of life decline [3,4,5,6]. The World Health Organisation defines rehabilitation as “a set of interventions designed to optimize functioning and reduce disability in individuals with health conditions in interaction with their environment” [7]. Functional status is critical when assessing the effectiveness of rehabilitation interventions as such interventions are organized and provided to ensure attainment of optimal functional improvement [8]. Therefore, the assessment of an individual’s functional status is central to the process of rehabilitation [9]. Despite widespread agreement that assessing functional status in older persons in rehabilitation is essential [10], the ideal method and preferred instruments for measuring health status, with a particular focus on functioning, is still uncertain. Several health status measures have been used in the rehabilitation of older people. These measures could be generic, such as the EuroQol 5 dimensions 3 level (EQ-5D-3L), or function-specific, such as the Barthel Index (BI) [11]. An attraction of the EQ-5D-3L is that responses to this instrument facilitate the calculation of quality adjusted life years (QALYs) required for conducting cost-utility analysis (CUA), a type of economic evaluation. It is the instrument of choice for performing CUA in some countries such as the UK [12]. Two recent systematic reviews [13,14] reported that it was one of the most widely used HRQoL instruments in economic evaluations of services for older people. An argument against using generic HRQoL instruments such as the EQ-5D-3L is that they can be insensitive and fail to adequately capture essential aspects of health status such as functioning [15]. When the goal is to measure functional status, function-specific instruments such as the BI are seen to be more sensitive [16]. The BI was initially developed to assess disability in patients with neuromuscular and musculoskeletal conditions who were receiving inpatient rehabilitation [11]. It is, however, now recommended for use in the routine assessment of older people by bodies such as the Royal College of Physicians and the British Geriatrics Society [17].

When considering health status measures for potential use in economic evaluation within rehabilitation research targeted at older people (where functional status is a major focus), it is unclear how the BI performs compared to the EQ-5D-3L in terms of construct validity. As the BI has been recommended as a proxy for HRQoL in the literature [18], evidence of a strong correlation between the BI and EQ-5D-3L and comparable or better discriminant validity would provide further evidence of the suitability of using the former for economic evaluations conducted within this population. Using data from a study that evaluated intermediate care services in the UK, this study assessed the convergent and divergent validity of the BI when compared against the EQ-5D-3L. Intermediate care comprises services, including rehabilitation, targeted at preventing admission to acute care or long-term care and aid discharge from hospital for older people in the UK [19]. Whereas the BI has been previously compared to the EQ-5D-3L in stroke patients [20], no simultaneous head-to-head comparison between the BI and the EQ-5D-3L has been performed in a more generic population of older people, as far as we are aware. Therefore, the results of this study will help inform decisions concerning the appropriateness of applying the BI within rehabilitation research focused on older people.

## 2. Materials and Methods

### 2.1. Study Design and Recruitment

This work is based on data collected from a national evaluation of intermediate care services for older people in the UK. Five anonymous primary care trusts (PCTs) were selected as case studies to represent intermediate care rehabilitation settings. A total of 2253 intermediate care participants were recruited from the five primary care trusts chosen to represent ‘whole systems’ (an area with a specific geographical boundary) of intermediate care [19,21]. By studying ‘whole systems’ instead of individual service models, we aimed to achieve a more detailed understanding of the implementation of intermediate care. The sites were included in the evaluation if they fulfilled the following criteria:

- A range of intermediate care services operational for at least 2–3 years.
- Reasonable throughput into the intermediate care system (at least 1000 cases per annum).
- A mix of urban and rural sites.
- Senior management support for the collection of routine data by services themselves.
- Clinical and managerial support for participation in the national evaluation.

Data were obtained from these clients at baseline and 12 months using a study proforma that included EQ-5D-3L and BI questionnaires. Other information collected included data on age, gender, living arrangements and type of rehabilitation services required (Table 1). In addition, whereas the EQ-5D-3L and BI questionnaires were completed by staff with or on behalf of their patients, only responses obtained using the former approach were included in this study for the sake of consistency in data collection methods. This study focuses only on respondents who received rehabilitation services and had complete data on both the BI and EQ-5D-3L at baseline. All participants provided written informed consent before their inclusion in the study. The Trent Multicentre Research Ethics Committee approved the study.

### 2.2. Instruments

#### 2.2.1. Barthel Index (BI)

The BI is a measure of functional status whose validity when used on a general population of older people has been shown [22]. The BI uses 10 items. Each item is weighted according to the professional judgement of the instrument developer with different weights to measure a person’s level of functional status (independence) [11]. Of the 10 items, two (bathing and grooming) are rated on a two-point scale of 0 and 1, six (feeding, dressing, bowels, bladder, toilet use and stairs) on a three-point scale of 0, 1 and 2 and the last two items (transfers and mobility) are rated on a four-point scale of 0, 1, 2 and 3. An overall score ranging from 0 to 20 (which can also be standardized to range between 0 and 100) is obtained by adding the scores on each item [11]. Higher BI overall and item scores represent a greater level of independence. The reliability, sensitivity and suitability for the use of the BI in older populations have been reported in the literature [23,24,25,26].

#### 2.2.2. EuroQol-5-Dimensions 3 Level (EQ-5D-3L)

The EQ-5D-3L is the three-level version of the EuroQol 5 Dimensions (EQ-5D) which is a standardized HRQoL questionnaire designed to produce a simple generic measure of health for use in clinical and economic appraisal for individuals aged ≥18 years. The EQ-5D-3L measures five dimensions: mobility, self-care, usual activities, pain/discomfort and anxiety/depression [27]. Each domain has three levels of impairment (‘no problems’, ‘some/moderate problems’ and ‘extreme’ problems), allowing the EQ-5D-3L to distinguish between 243 states of health [28]. Based on UK general population preference weights determined through the time trade-off approach [28], utilities ranging from −0.59 to 1 can be attached to each of the EQ-5D-3L health states. Higher utilities represent better HRQoL. The EQ-5D-3L has strong psychometric properties, and its validity, when used in populations of older people, has been proven [29,30,31,32].

### 2.3. Conceptual Overlap between Instruments and Hypotheses

To depict the conceptual overlap between these instruments, the dimensions were compared using the International Classification of Functioning, Disability and Health (ICF) Core Set framework [33]. The ICF is the most comprehensive attempt to classify health concepts within a biopsychosocial model of health, functioning and disability [33]. It has been linked to many patient-reported outcome development efforts, including that of the Patient-Reported Outcomes Measurement Information System (PROMIS) [34,35]. The instrument dimensions were linked to the ICF classification allowing them to be categorized into three potential domains, namely ‘body functions and structures’ (measuring impairments to (i) physiological and psychological functions of body systems and (ii) anatomical parts of the body such as limbs), ‘activities and participation’ (referring to constructs that cover the full range of life areas such as execution of tasks or actions and involvement in life situations) and ‘environmental factors’ (referring to the physical, social and attitudinal environment in which people live and conduct their lives which can be either barriers or facilitators to their functioning) [33]. Each of these domains was broken down into chapters, which were broken down further into categories (Table 1).

We, therefore, hypothesized that there would be moderate convergent validity between BI items and/or EQ-5D-3L dimensions that belonged to the same ICF domains. We also hypothesized that both instruments would discriminate between BI items and EQ-5D-3L dimensions that belonged to different ICF domains, i.e., the absolute correlation between these items and dimensions would be lower than that for items and dimensions belonging to the same ICF domains.

### 2.4. Statistical Analysis

Descriptive statistics of participants were estimated. The distribution of the BI and EQ-5D-3L was tested for normality using the Shapiro–Francia test (EQ-5D-3L: skewness = −1.030006, Kurtosis = 3.505952, z-score = 10.678, *p* < 0.001; BI: skewness = −1.197832, Kurtosis = 4.111406, z-score = 10.234, *p* < 0.001). Based on this test, appropriate non-parametric statistical tests of difference were applied.

Convergent validity evaluates the extent to which constructs that are expected to be related are, in fact, related [36]. The convergent validity of the BI item/total scores and EQ-5D-3L dimension/utility scores was assessed using several approaches. First, the level of association between BI item and EQ-5D-3L dimension scores was evaluated using Kendall’s Tau-B rank correlation coefficients, which have been recommended for ordinal categorical data [37]. We expected to see correlation between dimensions or items that theoretically belonged to the same ICF domains. Correlations below 0.30 were considered weak, those between 0.40 and 0.50 moderate and those above 0.50 strong (indicating that instruments were measuring similar constructs) [38]. Second, exploratory factor analysis (EFA) [39], incorporating polychoric correlation matrices, estimated using the robust weighted least squares approach, to accommodate the categorical nature of the instrument item/dimension responses [40,41], was undertaken. A decision to carry out EFA rather than confirmatory factor analysis (CFA) was driven by the fact that it was impossible to have a firm idea about the number of factors to be encountered and about which items/dimensions would most likely load onto each factor [39]. As recommended in the literature [42], only correlations ≥ 0.3 were considered to represent significant correlation in the EFA. The number of factors to include was determined by examining scree plots from the EFA. Due to this exercise’s exploratory nature, the EFA interpretation was based on a ‘varimax orthogonal rotated’ solution [43]. Convergence between the instruments was confirmed by BI items loading significantly onto the same factors as the EQ-5D-3L dimensions. Third, scatter plots between BI total and EQ-5D-3L utility scores were examined. The strength of convergence at the total/utility score level was further assessed by the size of Krippendorff’s alpha (estimating the levels of reliability between the instruments) [44] and the correlation coefficient (measuring the level of association between the instruments) [38]. An alpha of 0.80 or over-represented good agreement [45]. Lastly, modified Bland-Altman plots were also used to further study the limits of agreement between the two instruments. As recommended in the literature [46], standardized Z scores of utilities/total scores were calculated for the modified plots because the instruments use different rating scales leading to marked differences in the magnitude of the scores (i.e., the maximum BI total scores can be up to 169 times larger than those for the EQ-5D-3L). Utilities and total scores were power transformed to follow a normal distribution before calculating Z scores.

Campbell and Fiske [47] posit that different measures of the same hypothetical construct should correlate highly with one another if the measures are valid. In terms of convergent validity, therefore, we hypothesized, based on the content and descriptions of the instruments [11], that there would be a moderate to strong correlation between conceptually related items and dimensions, e.g., between the ‘mobility’ item of the BI and the ‘mobility dimension of the EQ-5D-3L. Specific hypotheses are presented in Table 2.

Discriminant validity assesses the extent to which constructs that theoretically should not be highly related to each other are, in fact, not found to be highly correlated to each other [48]. Two approaches were used to determine discriminant validity. Firstly, the correlation between EQ-5D-3L dimensions and BI items that theoretically did not belong to the same ICF domains was established. We expected discriminant validity coefficients to be smaller in magnitude than convergent validity coefficients [48]. Secondly, the discriminant ability of the instruments was tested using EFA by assessing whether BI items and EQ-5D-3L dimensions loaded more highly onto their own factor than to another or other factors. Cases where the variance extracted estimate (AVE) was greater than the squared correlation estimate (Corr^2^) were indicative of good discriminant validity [49].

For discriminant validity, we postulated that discriminant validity coefficients would be smaller in magnitude than convergent validity coefficients for both instruments. We also expected the BI items and EQ-5D-3L dimensions to load more highly onto their own factors due to differences in the health constructs they were designed to measure [27].

## 3. Results

### 3.1. Participant Characteristics

Of the 2253 who were recruited, 1690 had complete data for both BI and EQ-5D-3L. Characteristics for 1690 study respondents are presented in Table 2. The mean ± standard deviation (SD) values for the BI total and EQ-5D-3L utility scores were 79.320 ± 19.465 and 0.499 ± 0.351, respectively.

**Figure 1:**
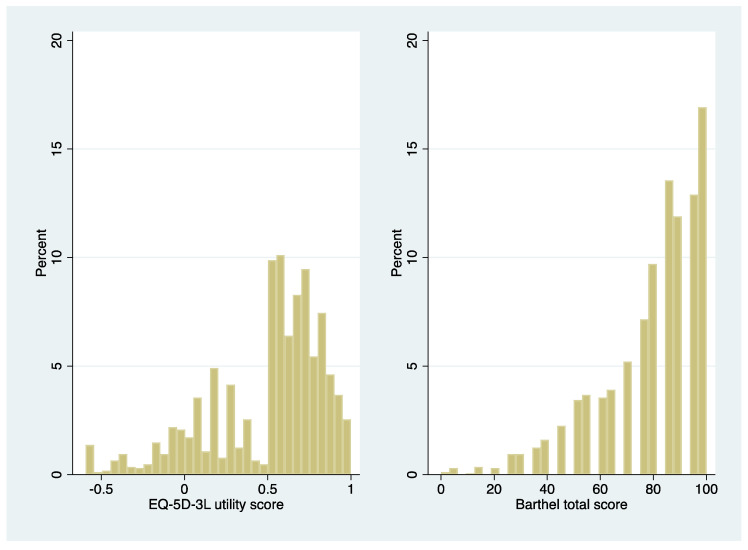
Presents the distribution of EuroQol 5 dimensions 3 levels (EQ-5D-3L) utilities and Barthel index (BI) total scores.

Both instruments had non-normal distributions (*p* < 0.0001, Shapiro–Francia test; Figure 1). Therefore, non-parametric tests of differences were applied to all analyses reported below.

### 3.2. Conceptual Overlap between Instruments

Analysis of the conceptual overlap of the EQ-5D-3L and BI revealed that the dimensions of both instruments capture domains related to ‘Body functions’ and ‘Activities and Participation’, as shown in Table 2. Two EQ-5D-3L dimensions (Anxiety/depression and Pain/discomfort) and two BI items (Bowels and Bladder) were classified under the same ICF domain of ‘Body Functions’, but a more detailed analysis showed that each of these four dimensions belonged to different chapters within this domain (Table 2). The most overlap was in terms of EQ-5D-3L dimensions (three out of five) and BI items (eight out of 10) that captured the ICF domain of ‘Activities and Participation’. The EQ-5D-3L dimension of ‘Mobility’ and the BI items of ‘Mobility’, ‘Stairs’ and ‘Transfer’ belonged to the ‘Mobility’ chapter of the ‘Activities and Participation’ domain. Further, the EQ-5D-3L dimension of ‘Self-Care’ and the BI items of ‘Bathing’, ‘Grooming’, ‘Feeding’, ‘Dressing’ and ‘Toilet use’ belonged to the ‘Self-Care’ chapter of the ‘Activities and Participation’ domain. The ‘usual activities’ dimension of the EQ-5D-3L could separately be potentially linked to the four ICF ‘Activities and Participation’ domain chapters: ‘Domestic Life’, ‘Interpersonal Interactions and Relationships’, ‘Major Life Areas’ and ‘Community, Social and Civic Life’. Overall, our analysis showed a considerable correlation between the two instruments as all EQ-5D-3L dimensions and BI items showed conceptual overlap at the ICF domain level. When the domains were broken down into chapters, our analysis showed conceptual overlap between two EQ-5D-3L dimensions and eight BI items.

### 3.3. Convergent Validity

Table 3 presents bivariate correlation coefficients, all statistically significant at a 5% significance level, depicting the relationship between BI item and EQ-5D-3L dimension scores. The results of this analysis show that there was weak to moderate statistically significant negative association (Spearman’s correlation, *p*-value < 0.001) between the two instruments ranging from −0.11 (‘Bathing’ (BI) versus ‘Pain/Discomfort’ (EQ-5D-3L)) and (‘Feeding’ (BI) versus ‘Anxiety/Depression’ (EQ-5D-3L)) to 0.54 (‘Dressing’ (BI) versus ‘Self-care’ (EQ-5D-3L)). As was hypothesized, correlations between BI items and EQ-5D-3L dimensions measuring similar constructs were in the direction expected, but some were weak rather than moderate. Our analysis shows that the absolute correlation between dimensions or items that theoretically belonged to the same ICF domains was on average 0.43 (between BI items), 0.41 (between EQ-5D-3L dimensions) and 0.36 (between EQ-5D-3L dimensions and BI items).

The results from the EFA shown in Table 4 depict the association between the items/dimensions of the instruments and latent or unobserved constructs. An examination of the scree plot (available from the author upon request) from this analysis suggested that extracting two factors was adequate. Table 4 shows that all BI items loaded more highly onto factor 1 (factor loadings ≥ 0.57), indicating that they are all associated with the latent variable or construct represented by this factor. However, only two EQ-5D-3L dimensions (‘self-care’ and ‘usual activities’) were also associated with this factor. Similarly, all EQ-5D-3L dimensions loaded more highly onto factor 2 (factor loadings ≥ 0.64) with only five BI items also loading highly onto this factor, i.e., ‘dressing’, ‘toilet use’, ‘transfers’, ‘mobility’ and ‘stairs’. There was some cross-loading in the EFA as well, with some BI items and EQ-5D-3L dimensions loading onto both factors.

**Figure 2:**
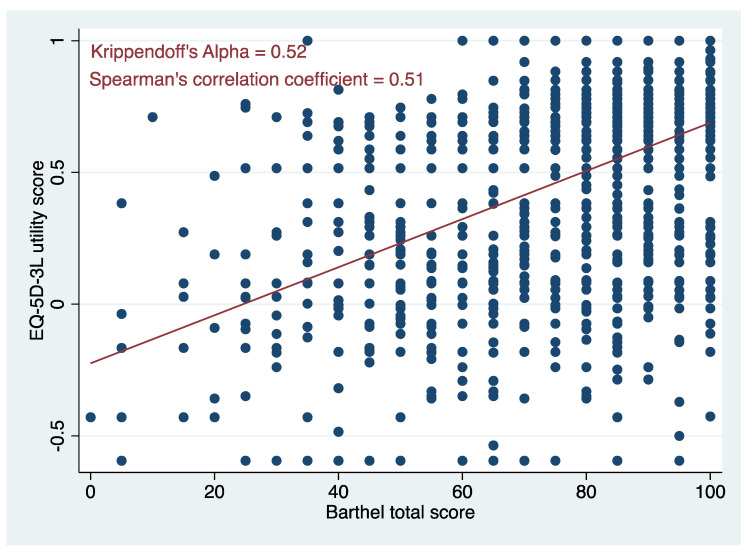
Shows a scatter plot between EuroQol 5 dimensions 3 levels (EQ-5D-3L) utilities and Barthel index total scores.

At the total/utility score level, a scatter plot between the two instruments (Figure 2) suggests a moderate correlation between them. This was confirmed by the size of Spearman’s correlation coefficient (r = 0.51, Figure 2) and Krippendorff’s alpha (0.52, Figure 2).

**Figure 3:**
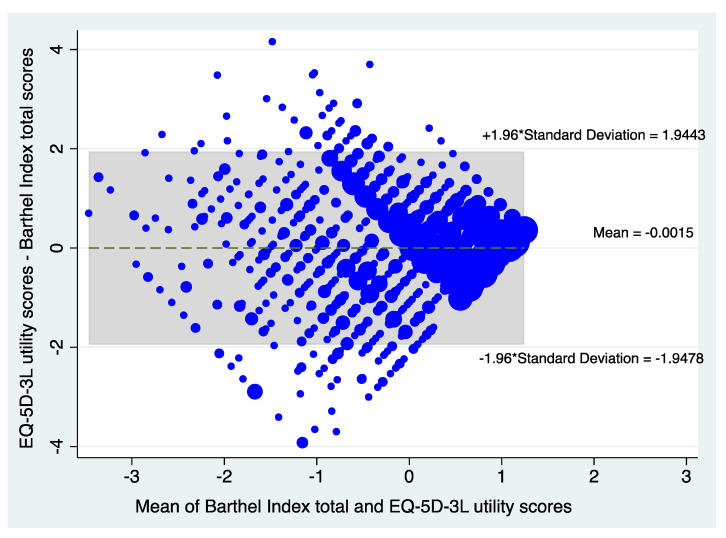
Presents the Bland and Altman Plot showing the level of agreement between the EuroQol 5 dimensions 3 levels (EQ-5D-3L) utility standardized Z scores versus Barthel index total standardized Z scores.

The modified Bland-Altman scatter plots in Figure 3 also suggest moderate agreement between the two instruments, with only about 6% of the Z scores outside the 95% limits of agreement.

### 3.4. Discriminant Validity

The results of this analysis (Table 3) show that the absolute correlation between dimensions or items that did not theoretically belong to the same ICF domains (‘Body Functions’ versus ‘Activities and Participation’) was on average 0.25 (between BI items), 0.30 (between EQ-5D-3L dimensions) and 0.22 (between EQ-5D-3L dimensions and BI items). These results also suggest that the BI had marginally better discriminant validity (lower discriminant validity coefficients) than the EQ-5D-3L. Overall, the discriminant validity coefficients were smaller in magnitude than the convergent validity coefficients.

The results of the EFA suggested that the BI items and EQ-5D-3L dimensions were able to discriminate between factors that they loaded onto as the estimate of the AVE (0.3791) was greater than that for Corr^2^ (0.0001).

## 4. Discussion

Using data from a population of older people receiving rehabilitation services, the present study reports on the first head-to-head empirical comparison between the BI, an instrument recommended for assessing functional status [11], and the EQ-5D-3L, a generic HRQoL instrument recommended for use within CUA [12]. When considering health status measures for potential use in economic evaluation within rehabilitation research targeted at older people, our analysis shows that the two instruments are both suitable as they were able to demonstrate discriminant validity. However, they need to be considered complements rather than substitutes for each other due to moderate convergence between them. The decision about which instrument to use and in which context should be guided by the ICF domain chapters the dimension or items load onto. If it is important to capture information on mental functions, sensory functions and pain, domestic tasks, interpersonal interactions and relationships, and community, social and civic life, then the EQ-5D-3L should be chosen. However, if it is more important to capture information on mobility and self-care, then the BI should be selected as it has more items capturing these two constructs than the EQ-5D-3L. An added consideration would be whether there is a desire for the economic evaluation results based on either instrument to be compared across sectors (i.e., compared to non-rehabilitation or aged care sectors using a CUA) or not. If the former is required, then the EQ-5D-3L would be the ideal measure and the BI if the latter is desired. Further, consideration should also be made to the descriptive basis of the two instruments as they differ in some respects with the scoring systems based on entirely different approaches, i.e., the BI being a scale with cardinal properties and the EQ-5D-3L being a semi-weighted ordinal scale. The EQ-5D-3L has been long established as the most widely used preference-based instrument worldwide. For instance, within economic evaluations, it is the most popular overall [43] and one of the most frequently used for services targeted at older people [13,14]. The development of a new five-level version of the instrument has improved its sensitivity and standardized the language across dimensions [51]. Similar to the EQ-5D-3L, the BI discriminated well between factors measuring latent constructs captured by the instrument and between dimensions of the EQ-5D-3L. Due to having discriminant validity comparable to that of the EQ-5D-3L, the BI would be equally valuable for measuring subgroup differences in studies where the former is routinely used, such as within economic evaluation. In economic evaluations and particularly in CEA, however, the BI can be used to measure non-utility-based functional status as it does not have utility weights.

As hypothesized, based on the ICF domains that BI and EQ-5D-3L items loaded on (Table 1), results from the bivariate correlation analysis and the EFA showed statistically significant convergence between items on the BI and EQ-5D-3L dimensions that measured similar constructs. However, the level of correlation was weak to moderate rather than moderate to strong. One explanation of these findings is the possibility that some pairwise correlations between items and dimensions were low because of unaccounted-for, unrecognized and unmeasured constructs that link them together that may not be adequately captured by the ICF framework [52]. For instance, it was possible that someone who can perform their ‘usual activities’ on the EQ-5D-3L may still not control their ‘bowels’ on the BI if they have a chronic bowel problem. Respondents may therefore view the lack of bowel control as ‘usual’ because they have adapted to a chronic condition. A similar finding was reported elsewhere [53]. Even though the BI items loaded highly onto different factors compared to the EQ-5D-3L dimensions in the EFA, there was sufficient overlap between some of these items and dimensions suggestive of moderate levels of agreement (>0.3). It is not surprising that the EFA suggested that the ‘self-care’ and ‘usual activities’ dimensions of the EQ-5D-3L measured similar latent constructs such as those of the BI. In an EFA comparing the EQ-5D-3L to the ICECAP-O, Keeley et al. [54] posited that these two EQ-5D-3L dimensions represent a single factor termed “physical functioning”, which is one of the critical constructs captured in the design of the BI. At the BI total/EQ-5D-3l utility score level, estimates of Spearman’s correlation coefficient and Krippendorff’s alpha, as well as an examination of the Bland-Altman plot, all suggest a moderate correlation between the instruments. This means that when all instrument items and dimensions are considered in their totality, they, on average, represent interrelated concepts. This has been shown in our other research [55,56,57].

The EFA showed that BI items and EQ-5D-3L dimensions each loaded more highly onto their own factor (factor 1 and 2, respectively; Table 3). Based on the dominance of items and dimensions that assess functional status associated with factor 1, we can characterize it as measuring ‘physical functioning’. We can further represent factor 2 as “wellbeing”, which has been suggested as a broader concept that captures the attributes such as those that loaded onto this factor [54]. An examination of the sizes of the factor loadings from the EFA suggests that these instruments should be seen as complements rather than substitutes for each other. This is because the only two EQ-5D-3L dimensions that loaded onto the ‘physical functioning’ factor (‘self-care’ and ‘usual activities’) did not load as highly as the BI items. Similarly, the five BI items that loaded onto the ‘wellbeing’ factor did not load as highly onto this factor as the EQ-5D-3L dimensions. This result supports the view that the BI can measure functioning-related aspects of health better than the generic HRQoL instrument. On the other hand, the size of the EQ-5D-3L loadings onto the ‘wellbeing’ factor suggest that this instrument can measure broader aspects of health better than the BI. This result has also been seen elsewhere [58].

Some limitations merit note. The analysis in this study focused on comparisons between two instruments collected using a cross-sectional study design. Therefore, future research should consider assessing the instruments’ responsiveness over time based on a longitudinal study design. Doing so will facilitate the evaluation of the capability of these instruments to detect clinically important changes over time in this population, a property important for assessing incremental effectiveness [59,60]. Additionally, consideration should also be given to extending the comparisons to other instruments designed for application with older people, such as the ICECAP-O, as these focus on quality-of-life constructs not presently captured in this study, such as individual capabilities [61,62].

## 5. Conclusions

There is no gold standard for measuring outcomes in populations of older people due to the heterogeneous impacts that conditions affecting them may have on health status, physical functioning and quality of life. As a result, an assessment of the convergent validity of one instrument when compared against another can only be implied rather than proved. However, our analysis shows that the BI has comparable discriminant validity, a property that is useful when conducting subgroup analysis. To capture more ‘activities-and-participation-specific ICF constructs not measured by a generic HRQoL instrument such as the EQ-5D-3L, the BI can be used within rehabilitation research. In cases where the focus is on capturing information on more ‘body-function-specific’ ICF constructs, then the BI should be selected as it has more items capturing this construct than the EQ-5D-3L.

## Figures and Tables

**Table 1:** Classifying EQ-5D-3L and Barthel Index dimensions according to the International Classification of Functioning, Disability and Health (ICF) classifications ^a^.

International Classification of Functioning, Disability and Health (ICF) Classifications			Instrument Dimensions	
Domains	ICF Chapter	Specific ICF Chapters and Categories	EQ-5D-3L ^a^	Barthel Index
Body Functions	Mental Functions Sensory Functions and Pain Voice and Speech Functions Functions of the Cardiovascular, Haematological, Immunological and Respiratory Systems Functions of the Digestive, Metabolic, Endocrine Systems Genitourinary and Reproductive Functions Neuromusculoskeletal and Movement-Related Functions Functions of the Skin and Related Structures	*Chapter: Mental Functions* ○ b152 Emotional functions ○ b156 Perceptual functions ○ b160 Thought functions	Anxiety/ depression	
		*Chapter: Sensory Functions and Pain* ○ b280 Sensation of pain ○ b298 Sensory functions and pain, other specified ○ b299 Sensory functions and pain, unspecified	Pain/ discomfort	
		*Chapter: Functions of the Digestive, Metabolic, Endocrine Systems* ○ b525 Defecation functions		Bowels
		*Chapter: Functions of the Digestive, Metabolic, Endocrine Systems* ○ b610 Urinary excretory functions ○ b6101 Collection of urine ○ b620 Urination functions ○ b6200 Urination ○ b630 Sensations associated with urinary functions ○ e1151 Assistive products and technology for personal use in daily living s570 Structure of gall bladder and ducts ○ s6102 Urinary bladder		Bladder
Body structure	Structure of the Nervous System The Eye, Ear and Related Structures Structures Involved in Voice and Speech Structure of the Cardiovascular, Immunological and Respiratory Systems Structures Related to the Digestive, Metabolic and Endocrine Systems Structure Related to Genitourinary and Reproductive Systems Structure Related to Movement Skin and Related Structures	N/A	N/A	N/A
Activities and Participation	Learning and Applying Knowledge General Tasks and Demands Communication Mobility Self-Care Domestic Life Interpersonal Interactions and Relationships Major Life Areas Community, Social and Civic Life	*Chapter: Mobility* ○ d410–d429 Changing and maintaining body position ○ d450–d469 Walking and moving ○ d498 Mobility, other specified ○ d499 Mobility, unspecified	Mobility	Mobility Stairs Transfer
		*Chapter: Self-Care* ○ d510 Washing oneself ○ d520 Caring for body parts ○ d530 Toileting ○ d540 Dressing ○ d550 Eating ○ d560 Drinking ○ d570 Looking after one’s health ○ d598 Self-care, other specified ○ d599 Self-care, unspecified	Self-care	Bathing Grooming Feeding Dressing Toilet use
		*Chapter: Domestic* ○ d610–d629 Acquisition of necessities ○ d630–d649 Household tasks ○ d630–d649 Household tasks ○ d650–d669 Caring for household objects and assisting others ○ d650–d669 Caring for household objects and assisting others ○ d698 Domestic life, other specified ○ d699 Domestic life, unspecified	Usual activities	
		*Chapter: Interpersonal Interactions and Relationships* ○ d710–d729 General interpersonal interactions ○ d710 Basic interpersonal interactions ○ d720 Complex interpersonal interactions ○ d729 General interpersonal interactions, other specified and unspecified ○ d730–d779 Particular interpersonal relationships ○ d798 Interpersonal interactions and relationships, other specified ○ d799 Interpersonal interactions and relationships, unspecified	Usual activities	
		*Chapter: Major Life Areas* ○ d810–d839 Education ○ d840–d859 Work and employment ○ d860–d879 Economic life ○ d898 Major life areas, other specified ○ d899 Major life areas, unspecified	Usual activities	
		*Chapter: Community, Social and Civic Life* ○ d910 Community life ○ d920 Recreation and leisure ○ d930 Religion and spirituality ○ d940 Human rights ○ d950 Political life and citizenship ○ d998 Community, social and civic life, other specified ○ d999 Community, social and civic life, unspecified	Usual activities	
Environmental Factors	Products and Technology Natural Environment and Human-Made Changes to Environment Support and Relationships Attitudes Services, Systems and Policies	N/A	N/A	N/A

^a^ EQ-5D-3L = EuroQol 5 dimensions 3 levels; SF6D = Short Form 6 dimensions.

**Table 2:** Baseline characteristics of the study participants.

Characteristic ^a^	Mean (SD) ^b^	Median (IQR) ^b^
Age	78 (11)	80 (73, 86)
EQ-5D-3L utility score	0.499 (0.351)	0.587 (0.26, 0.743)
BI total score	79.32 (19.465)	85 (70, 95)
EQ-5D-3L utility Z-score ^c^	−0.002 (0.999)	0.248 (−0.684, 0.692)
BI total Z-score ^c^	0.001 (1)	0.292 (−0.479, 0.806)
**Characteristic**	***n* (%)**	
Gender		
Male	515 (30)	
Female	1175 (70)	
Living Arrangements		
No	748 (44)	
Yes	942 (56)	
Type of Intermediate care rehabilitation service ^d^		
Acute Admission avoidance	659 (39)	
Supported Discharge	961 (57)	
Residential	70 (4)	

^a^ Total *n* = 1690; EQ-5D-3L = EuroQol 5 Dimensions 3 Levels. BI = Barthel Index. ^b^ IQR = Interquartile range. SD = Standard deviation. ^c^ EQ-5D-3L utility scores and Barthel total were first power transformed to follow a normal distribution (using the square transformation) before converting them into Z scores. ^d^ Only patients seeking rehabilitation services were included.

**Table 3:** Relationship between the descriptive classifications of the EuroQoL 5 dimensions 3 levels (EQ-5D-3L) and dimension and the item scores of the Barthel Index (BI) ^a^: Kendall’s Tau-B rank correlation coefficients showing correlation between the EQ-5D-3L dimension and BI item scores ^a^.

		EQ-5D-3L ^b^ Dimensions					Barthel ^b^ Items									
		Mobility	Self-Care	Usual Activities	Pain/Discomfort	Anxiety/Depression	Feeding	Bathing	Grooming	Dressing	Bowels	Bladder	Toilet Use	Transfers	Mobility	Stairs
**EQ-5D-3L Dimensions**	Mobility	-					−0.17	−0.20	−0.22	−0.25	−0.14	−0.16	−0.28	−0.31	−0.31	−0.28
	Self-care	0.45	-				−0.29	−0.35	−0.42	−0.54	−0.17	−0.20	−0.39	−0.40	−0.36	−0.29
	Usual activities	0.39	0.46	-			−0.22	−0.29	−0.28	−0.37	−0.09	−0.12	−0.30	−0.32	−0.30	−0.26
	Pain	0.35	0.27	0.28	-		−0.12	−0.11	−0.14	−0.17	−0.06	−0.06	−0.16	−0.20	−0.19	−0.17
	Anxiety	0.28	0.32	0.28	0.32	-	−0.11	−0.16	−0.20	−0.24	−0.11	−0.14	−0.24	−0.25	−0.26	−0.15
**Barthel Index Items**	Feeding						-									
	Bathing						0.21	-								
	Grooming						0.38	0.38	-							
	Dressing						0.40	0.42	0.64	-						
	Bowels						0.19	0.10	0.24	0.19	-					
	Bladder						0.24	0.18	0.32	0.31	0.38	-				
	Toilet use						0.44	0.30	0.53	0.55	0.26	0.35	-			
	Transfers						0.40	0.28	0.50	0.54	0.26	0.34	0.74	-		
	Mobility						0.36	0.28	0.46	0.50	0.24	0.32	0.70	0.74	-	
	Stairs						0.18	0.40	0.33	0.38	0.19	0.22	0.34	0.34	0.38	-

^a^ All correlations were statistically significant. Underlined correlation coefficients indicate that at least moderate absolute correlation between dimension and item scores were hypothesized based on the fact that these dimensions and items belonged to the same International Classification of Functioning, Disability and Health (ICF) domains [33]. Note that dimension scores for the EQ-5D-3L are ‘reverse-scored’ so that a higher (lower) score implies lower (higher) health-related quality of life. ^b^ EQ-5D-3L = EuroQol 5 Dimensions 3 Levels. Barthel = Barthel Index.

**Table 4:** Results of the exploratory factor analysis showing which factors the EuroQoL 5 dimensions 3 levels (EQ-5D-3L) dimensions and the Barthel Index (BI) items loaded onto ^a^.

Variable	Factor1	Factor2	Uniqueness
Barthel Index Dimensions			
Feeding	0.6565		0.5210
Bathing	0.6571		0.4804
Grooming	0.8207		0.2606
Dressing	0.7982	−0.3522	0.2388
Bowels	0.5767		0.6592
Bladder	0.6418		0.5853
Toilet use	0.8563	−0.3604	0.1369
Transfers	0.7934	−0.4303	0.1853
Mobility	0.7433	−0.4334	0.2597
Stairs	0.5859	−0.3066	0.5628
EQ-5D-3L ^b^ dimensions			
Mobility		0.7277	0.3899
Self-care	−0.4931	0.6432	0.3431
Usual activities	−0.3105	0.6496	0.4816
Pain/Discomfort		0.6402	0.5871
Anxiety/Depression		0.5723	0.6431
Variance explained by factors	64.96%	35.04%	
Average variance extracted (AVE) ^c^	0.3791		
Correlation between factors	0.0001		
Determinant	0.003		
Bartlett test of sphericity	χ^2^ = 9781.303, *p* < 0.001		
Kaiser-Meyer-Olkin Measure ^d^	0.899		

^a^ Blanks represent absolute(loading) < 0.3, which was threshold assumed for significant correlation [42]. ^b^ EQ-5D-3L = EuroQol 5 Dimensions 3 Levels. ^c^ The AVE measures the average amount of variance that a construct explains in its indicator variables (dimensions and items of the EuroQoL 5 dimensions 3 levels and Barthel index instruments, respectively) relative to the overall variance of all these indicators. The AVE was calculated using the formula recommended by Gefen et al. [50]. ^d^ Kaiser-Meyer-Olkin Measure of Sampling Adequacy.

## Data Availability

Data used in this study are available from the authors upon request.
